# Human Neutrophil Peptide 1 as immunotherapeutic agent against *Leishmania* infected BALB/c mice

**DOI:** 10.1371/journal.pntd.0006123

**Published:** 2017-12-18

**Authors:** Zahra Abdossamadi, Negar Seyed, Farnaz Zahedifard, Tahereh Taheri, Yasaman Taslimi, Hossein Montakhab-Yeganeh, Alireza Badirzadeh, Mohammad Vasei, Safoora Gharibzadeh, Sima Rafati

**Affiliations:** 1 Department of Immunotherapy and *Leishmania* Vaccine Research, Pasteur institute of Iran, Tehran, Iran; 2 Cell-Based Therapies Research Center, Digestive Disease Research Institute and Department of Pathology, Shariati Hospital, Tehran University of Medical Science, Tehran, Iran; 3 Department of Epidemiology and Biostatistics, Pasteur Institute of Iran, Tehran, Iran; Northeastern University, UNITED STATES

## Abstract

Human Neutrophil Peptide 1 (HNP1) produced by neutrophils, is a well-known antimicrobial peptide which plays a role both in innate as well as in adaptive immunity and is under intensive investigation as a potential therapeutic agent. Previous *in vitro* experiments have indicated the leishmaniacidal effect of recombinant HNP1 on *Leishmania major* (*L*. *major*) promastigotes and amastigotes. In the current study, we further extended the idea to explore the remedial effect of HNP1 in the two modalities of peptide therapy (folded HNP1) and gene therapy in *L*. *major* infected BALB/c mice. To this end, mice in five different groups received synthetic folded HNP1 (G1), pcDNA-HNP1-EGFP (G2), pcDNA-EGFP (G3), Amphotericin B (G4) and PBS (G5), which was started three weeks after infection for three consecutive weeks. Footpad swelling was monitored weekly and a day after the therapy ended, IFN-γ, IL-4, IL-10, IL-6 and nitric oxide produced by splenocytes were analyzed together with the parasite load in draining lymph nodes. Arginase activity and dermal histopathological changes were also analyzed in the infected footpads. We demonstrated that both therapeutic approaches effectively induced Th1 polarization and restricted parasite burden. It can control disease progression in contrast to non-treated groups. However, pcDNA-HNP1-EGFP is more promising in respect to parasite control than folded HNP1, but less effective than AmB treatment. We concluded with the call for a future approach, that is, a DNA-based expression of HNP1 combined with AmB as it can improve the leishmaniacidal efficacy.

## Author summary

The outbreak level of cutaneous leishmaniasis is approximated between one and 1.5 million individuals per year. Owning to several disadvantages of current therapies, special attention to expand novel and efficient therapies has been demanded. Among Anti-Microbial Peptides (AMPs), Human Neutrophil Peptide 1 (HNP1) is one of the most potential defensins. Our promising *in vitro* experiments have shown the leishmaniacidal effect of recombinant HNP1. Here, we displayed the remedial effect of HNP1 in two approaches including peptide therapy and gene therapy in susceptible mice infected with *L*. *major*. Our investigation showed that although both approaches could decrease the parasite load and induce Th1 immune response compared to the control group, pcDNA-HNP1-EGFP has a better effect compared to the folded HNP1. Hence, immunotherapy by HNP1 can help elicit proper immunity despite the direct effect on promastigotes and amastigotes forms of parasite.

## Introduction

Leishmaniasis is as an important neglected tropical disease in poverty-stricken countries of Africa, South America, and Asia [1,2]. Leishmaniasis has three major manifestations: Visceral leishmaniasis, cutaneous leishmaniasis and mucocutaneous leishmaniasis [3]. Considering the complex life cycle including promastigote (as an extracellular flagellated form) and amastigote (as an intracellular aflagellated form) with differential protein expression, parasite elimination is a hard task and controlling the disease is difficult [4]. Disease control still relies on chemotherapy and an effective prophylactic vaccine is not yet available. Nevertheless, disadvantages of chemotherapy including toxicity and vigorous harmful response have restrained their use [5–8], and instead a great deal of attention is drawn by new immunotherapeutic agents to promote the immune response [9]. Some studies described the *in vitro* leishmaniacidal effect of AMPs [4,10–12]. In this respect, defensins derived from different innate cells like neutrophils [13] are extremely attractive immunomodulators with promising antibacterial, anti-viral, leishmaniacidal and anti-cancer effect [9,14–16]. In particular, defensins and cathelicidins are among the intensely investigated antimicrobial peptides [17–19].

Our previous *in vitro* results clearly demonstrated the anti-leishmanial effect of recombinant HNP1 against *L*. *major* promastigotes and amastigotes [15]. Due to limited knowledge about the effect of Anti-Microbial Peptides (AMPs) on leishmaniasis, we further expanded the idea to examine the therapeutic effect of HNP1 to treat infected BALB/c mice with *L*. *major*.

We compared the therapeutic effect of the two different approaches of HNP1 as folded synthetic peptide and pcDNA-HNP1-EGFP with the AmB treatment as the current standard therapy. We demonstrated that HNP1 is a potential molecule that can both modulate the immune response and kill the parasite. So, it has the capacity to contribute as an anti-leishmaniacidal agent for the *in vivo* condition in susceptible BALB/c mice and could further expand the idea as mixed chemo- immunotherapy formulations in the near future.

## Materials and methods

### Chemicals, media and reagents

All solutions were prepared with apyrogenic deionized water (MilliQSystem (Millipore, Molsheim, France)). The required materials for PCR reaction, enzymatic digestion and agarose gel electrophoresis were purchased from Roche Applied Sciences (Germany). Bovine Serum Albumin (BSA), Diaminobenzidine (DAB) powder and acrylamide were from Merck company (Germany). G418, Sodium dodecyl sulfate (SDS), Urea, Tris-HCL, Tris-base and Ponseu-S, Phorbol 12 myristate 13-acetate (PMA), and 3-(4,5 dimethylthiazol-2-yl)-2,5-diphenyl tetrazolium bromide (MTT), M199 medium, L-glutamine, HEPES, adenosine, hemin, gentamicin and RPMI-1640, DMEM (used for cell culture) and kanamycin were purchased from Sigma (Germany). Schneider insect media and Fetal Calf Serum (FCS) were from Gibco (Gibco, Life Technologies, Germany) and Sigma (Germany), respectively. Amphotericin B (AmB) was purchased (Cipla, India).

### Ethical aspects

All mice experiments including maintenance, handling and euthanizing were authorized by Institutional Animal Care and Research Advisory Committee of Pasteur Institute of Iran with ethical code IR.RII.REC.1395.55 (dated 2014), based on the Specific National Ethical Guidelines for Biochemical Research issued in 2005 by the Research and Technology Deputy of Ministry of Health and Medicinal Education (MOHM) of Iran. In this study, mice were euthanized through cervical dislocation by a trained and experienced technician. The efforts were made to minimize animal suffering within the course of our study.

### Cloning pathway for constructs used in gene therapy

The HNP1 gene was amplified from pcDNA3.1(+) vector (for harboring Kozak sequence and *Sal*I and *Bam*HI restriction sites) with two forward (F1; 5'-GTCGACACCATGGCCTGCTATTGC-3', F2; 5'-CCATCTGGCTAGCGTCGACACCAT-3') and reverse (R; 5'- GATGGATCCGCAGCAGAATGCCCAG-3') primers with Taq DNA polymerase and was cloned in *Nhe*I, *Bam*HI sites in pcDNA 3.1(+) and pGEM7zf(-) vectors (Invitrogen, Germany) and sequenced. After sequence validation, the HNP1 fragment was subcloned into the *Nhe*I and *Bam*HI sites from pEGFP.N3 vector for harboring EGFP and then HNP1-EGFP was subcloned into *Nhe*I site from pcDNA3.1(+) vector for providing pcDNA-HNP1-EGFP as a therapeutic construct. Also EGFP was subcloned into *Bam*HI and *Not*I cloning sites in pcDNA3.1(+) vector for creating pcDNA-EGFP as a control in therapeutic process. GFP expression in both constructs was confirmed in COS-7 cell line and plasmids were extracted in large scale using Endo free Mega kit (QIAGEN, Germany).

### COS-7 cell transfection with PEI treatment

The COS-7 cells (ATCC TIB-202) were used for expression confirmation of HNP1-EGFP in pcDNA-HNP1-EGFP. Briefly, COS-7 cells were cultured at 37°C in the presence of 5% CO_2_ and then transfected with PEI/DNA complexes prepared by mixing LINPEI (7 μM) with 5 μg of each pcDNA-HNP1-EGFP, and pcDNA-EGFP (as control) [20]. 48h later fluorescent cells were assessed by fluorescence microscopy (Nicon E200, Japan), and were also subject to western blotting with monoclonal anti GFP antibody.

### Western blot analysis of gene expression

The pcDNA-HNP1-EGFP and pcDNA-EGFP transfected COS-7 cells were trypsinized and harvested. Cell pellets, were boiled while mixed with 2X SDS PAGE sample buffer (4.5Mm Tris-Hcl, PH 6.8, 10% v/v glycerol, 2% w/v SDS, 5% v/v 2-mercapthoethanol, 0.05 w/v bromophenol blue), and were loaded on a 17.5% polyacrylamide gel (SDS gel apparatus; Bio Rad). The isolated bands were transferred from SDS-PAGE gel onto Protran nitrocellulose membrane through a wet blotting system (Bio-Rad, USA) after blocking (PBS with 2.5% BSA and 0.1% Tween) for overnight at 4°C. The EGFP bands were developed after blotting with anti-GFP antibody conjugated with horse radish peroxidase diluted 1:5000 (Acris Antibodies GmbH, Herford, Germany) and Diaminobenzidine (DAB) as chromophor.

### *In vitro* folding of synthetic HNP1 and function assay of folded HNP1 using wild type *E*. *coli* bacteria and *L*. *major* promastigotes

HNP1 was synthesized with a purity of >90% (93.17%) by Biomatik (Cambridge, Canada) (S1 Fig) and then was folded by oxido-shuffling process [21]. Briefly, the purified HNP1 peptide at the concentration of 160 μg/ml was solubilized in folding buffer (Tris–HCL 50mM, NaCl 100mM, EDTA 0.1mM, reduced glutathione 3mM and oxidized glutathione 0.3 mM, pH 8.5) and kept at 4°C in rotator (40 rpm) for overnight, then diluted in 10X volume of changing buffer and centrifuged in 3000 × g at 4°C for 30 min to precipitate aggregates.

The antibacterial effect was examined in comparison with unfolded HNP1 on *E*. *coli* (gift strain from Microbiology Dept. Pasteur Institute of Iran). The antibacterial activity was analyzed according to established protocol by Paziger and Lubkowski [22]. Briefly, the mid log phase (optical density 0.4–0.5 at 600 nm absorbance) of wild type *E*. *coli* was diluted in changing buffer (Tris-CL 10 Mm, Nacl 100 mM) to 10^6^ Colony Forming Unit (CFU; viable number of bacteria in sample). Then, different concentration of folded HNP1 (2.5 μg/ml to 40 μg/ml; two fold serial dilution) were used and incubated for 3h at 37°C. Finally, the serially diluted and cultured on LB agar plates for 24h at 37°C. Each concentration of HNP1 at least repeated in three independent experiments and reported in CFU/ml of bacteria culture.

The efficacy of folded HNP1 with different concentrations from 5μg/ml to 35μg/ml for leishmaniacidal effect of 2×10^6^ logarithmic phase promastigotes was assessed in comparison with AmB (0.15 μg/ml to 2.5 μg/ml; two fold serial dilution) and susceptibility of selected concentration were analyzed by MTT assay [23], followed by determination of IC_50_ (the concentration of HNP1 and AmB where the viable promastigotes growth is reduced by half).

### Parasite rescue assay and determination of EC_50_

The THP-1 cells were pretreated with PMA to form adherent cells and incubated at 37°C, for efficacy evaluation of folded HNP1 on *L*. *major* infected cells using parasite rescue assay [24]. Briefly, 5×10^6^ *L*. *major* promastigotes at stationary phase was incubated with 5×10^5^ human macrophage THP-1 at a ratio of 10 parasites to 1 THP-1 cells. After 24h, extracellular parasites were washed out, then different concentrations of folded HNP1 (17 μg/ml to 53 μg/ml) were added. Meanwhile, AmB (0.15 μg/ml to 2.5μg/ml; two fold serial dilution) was used as positive control. After 48h, amastigotes were released by 0.05% SDS treatment and Schneider medium contained 10% inactivated FCS was added. Plates were incubated at 26°C for amastigote to promastigote transformation. Eventually, the EC_50_ (the concentration of HNP1 and AmB where the viable amastigotes growth is reduced by half) was determined using MTT assay [23].

### Dose determination of folded HNP1 peptide

Female BALB/c mice (n = 3) were infected by IP injection of stationary phase *L*. *major* promastigotes. Total of 5 × 10^8^ in 500 μL serum free RPMI per mouse was administrated. Twenty four hours after infection, animals were treated with different concentration of HNP1 (30, 60 and 90 μg/ml) and two control groups of animals were also assigned as mice treated with Amphotericin B (8 mg/kg) and infected mice without any treatment (non-treated). After 24 hrs, parasite rescue assay and MTT test were assessed as described earlier.

### Mice infection

Six weeks old female BALB/c mice with an average weight of 20 g each (20 ± 3 g) were purchased from the breeding stock facilities at the Pasteur Institute of Iran. All mice were maintained under standard condition in proper cages, standard condition of light and diet. *L*. *major* (MRHO/IR/75/ER) was isolated from lymph node of infected mice and transferred to M199 media supplemented with10% FCS. Stationary phase metacyclic promastigotes were harvested on Ficoll 400 as described previously [24]. Then 2×10^6^ parasites in 50μl PBS (1X) buffer were injected to left hind footpad. Furthermore, the same parasite culture (10^8^ parasites/ml) was used for Frozen and Thawed (F/T) antigen preparation in liquid nitrogen and 37°C water bath, respectively [25] and protein concentration was further determined by bicinchoninic acid (BCA, PIERCE) test.

### Treatment schedule

Three weeks after infection, treatment was started for all five study groups (15 mice per group). Group 1 (G1) received folded HNP1 (30 μg/mouse) (S2 Fig), Group 2 (G2) received pcDNA-HNP1-EGFP (50 μg /mouse), Group 3 (G3) received pcDNA-EGFP (50 μg /mouse), Group 4 (G4) received AmB (8mg/ kg), and Group 5 (G5) received PBS (50μl). All the injections were carried in footpad at three consecutive weeks except than AmB which was injected intra peritoneal, for ten consecutive days (Table 1). Starting one week after infection, footpad swelling was monitored weekly until six weeks post infection by means of metric caliper. Swelling size was reported as the thickness of injected footpad after subtraction of the non-injected footpad as control.

**Table 1:** Routes, timelines and therapeutic doses in different groups.

Groups	Route of injection	Amount of injection	Number of injection	Duration of treatment
**G1 (Folded HNP1)**	s.c.*	30μg/mouse	Once a week	Three weeks
**G2 (pcDNA-HNP1-EGFP)**	s.c.	50μg/50μl	Twice a week	Three weeks
**G3 (pcDNA- EGFP)**	s.c.	50μg/50μl	Twice a week	Three weeks
**G4 (AmB)**	i.p.**	8mg/kg	10 days	10 days
**G5 (PBS**	s.c.	50 μl	Twice a week	Three weeks

*s.c: sub cutaneous

**i.p: intra peritoneal

### Lymph node parasite quantification by real time PCR

Six weeks after infection, five mice of each group were sacrificed, then the lymph nodes were excised and homogenized. Genomic DNA extraction was carried out using manufacture's instruction (GF-1 kit Vivantis, Malaysia). DNA concentration was measured by Nanodrap (Nanodrop, ND-1000, USA) and real time PCR (Applied Biosystem 7500) was fulfilled based on absolute copy number by two sets of primers for kinetoplastid minicircle region named RV1 (forward: 5'-CTTTTCTGGTCCCGCGGGTAGG-3'), and RV2 (reverse: 5'-CCACCTGGCCTATTTTACACCA-3') [26]. To draw standard curve, genomic DNA of 3×10^7^ *L*. *major* parasites was used in 10 fold dilution up to seven folds. For quantification of parasite in lymph nodes, 30 ng of extracted DNA was used in RT-PCR reaction including 5 pmol of each primers, 1μM Quantifast SYBR Green (Qiagen, Germany) master mix in a final volume 20 μl. PCR program were as follows: 95°C for 5 min; 40 cycles containing from 95°C for 15 s, 58°C for 30 s, and 72°C for 40 s.

### Cytokine assay

Level of IFN-γ, IL-4, IL-10 and IL-6 were measured six weeks after infection in different groups. For this purpose, individual spleens of five mice in each group were treated with ACK lysis buffer (NH_4_CL 0.15M, KHCO_3_ 1mM, Na_2_EDTA 0.1mM) to remove erythrocytes of splenocyte suspension. Then, 3.5 × 10^6^ RBC free splenocytes were seeded per well in 48 culture plate and stimulated with *L*. *major* F/T Ag (10 μg/ml), or concanavalin A (Con A, 5μg/ml) as positive control. Plates incubated at 37°C and 5% CO2. The supernatants were collected after 24 and 72h for evaluation of IL-6 and IL-4 levels, respectively. Meanwhile supernatants collected after five days were analyzed for IL-10 and IFN-γ production. Cytokine assay by ELISA kit was according to manufacturer's instructions (DuoSet, R&D System). Briefly, ELISA plates were coated with capture antibody (each pertinent cytokine antibody) and incubated overnight at room temperature (RT). After washing, plates were blocked by 1% BSA at RT. Then dilutions of standard and supernatants in reagent diluent were added as duplicate. After two hours, the plates were washed and were incubated with detection antibody (biotin conjugated anti-cytokine Ab). Finally, the reaction was revealed by adding streptavidin conjugated Horse Radish Peroxidase (HRP) and then peroxidase substrate system (KPL, ABTS). The optical density (OD) was measured at 405nm with ELISA reader (TECAN, USA) and calculated with concentration according to standard curve.

### Measurement of arginase activity in infected footpad

In this study, the injected footpad was cut into two different pieces. One piece from five mice in each group was homogenized for further arginase activity assay. The arginase activity was followed as previously described [27]. Briefly, 25 μl of supernatant from each homogenized footpad after centrifugation was mixed with lysis buffer (0.1% Tris–HCL 50 μM, MnCl_2_ 100 μM Triton 0.1%) and were incubated for 7 min at 56°C to activate arginase. Arginine hydrolysis was carried out by incubating L-arginine (0.5 M) with mentioned lysate at 37°C for 60 min. The reaction was stopped in presence of H_2_SO_4_, H_3_PO_4_, and H_2_O (1/ 3/7, v/v/v). After adding isonitrosopropiophenone (ISPF, Sigma) and heating at 100°C for 45 min, Urea concentration as the product of arginase activity on arginine substrate was determined at 540 nm using an automatic micro plate spectrophotometer (TECAN, USA). One unite is defined as the amount of enzyme that hydrolyze the formation of one μM of urea per minute [28,29].

### Nitric Oxide (NO) analysis

Nitrite production by F/T Ag re-stimulated splenocyte was analyzed in supernatants (n = 5) collected after five days. In summary, same volume of supernatant was mixed with Griess reagent [0.1N (1-napthyl) ethylenediamine dihydrochloride, 1% sulfanil amide in 5% H_3_PO_4_] and incubated for 10 min at RT. Then OD was measured at 550 nm with ELISA reader (TECAN, USA) and the concentration was determined using standard curve obtained from sodium nitrite serial dilution (1–300 μM).

### Histopathological examination

In this study, the injected footpad was cut into two different pieces. One piece from two mice in each group was fixed by 10% formalin. After dehydration by increasing ethanol (from 70 to 100%), paraffin blocks were prepared (4 mm) and stained by Hematoxylin and Eosin (H&E). Dermal histopathological changes were analyzed in a blind manner. The presence of parasite, plasma cell, and granuloma were assessed in different scales; Not Seen (NS), 1+ (rare; occasionally seen in high-power-field), 2+ (minor; easily seen in each HPF), 3+ (moderate; easily seen in low power field) and 4+ (severe; full of cells in each field). In addition, the degree of inflammation was evaluated as limited or diffused.

### Statistical analysis

Mean (standard deviation: SD) values for normal variables and median (interquartile range) for skewed variables were reported based on their group status. Data among five groups were compared using one-way ANOVA and Kruskal-Wallis based on distribution of the variables. Pairwise Comparison of characteristics between participants in various groups was fulfilled by student’s t-test for normal variables and Mann-Whitney U test for skewed variables. Bonferroni correction was performed in case of multiple comparison tests (i.e. we adjusted significance level as α/n, n is equal to number of comparison). All P-values were two-tailed and those smaller than 0.05 were considered statistically significant. Statistical analyses carry out using software Graph-Pad Prism (Inc. 2007, San Diago, USA). Meanwhile, all indicated data are representative of two rounds of experiment.

## Results

### HNP1-EGFP expression in COS-7 cells

**Fig 1:**
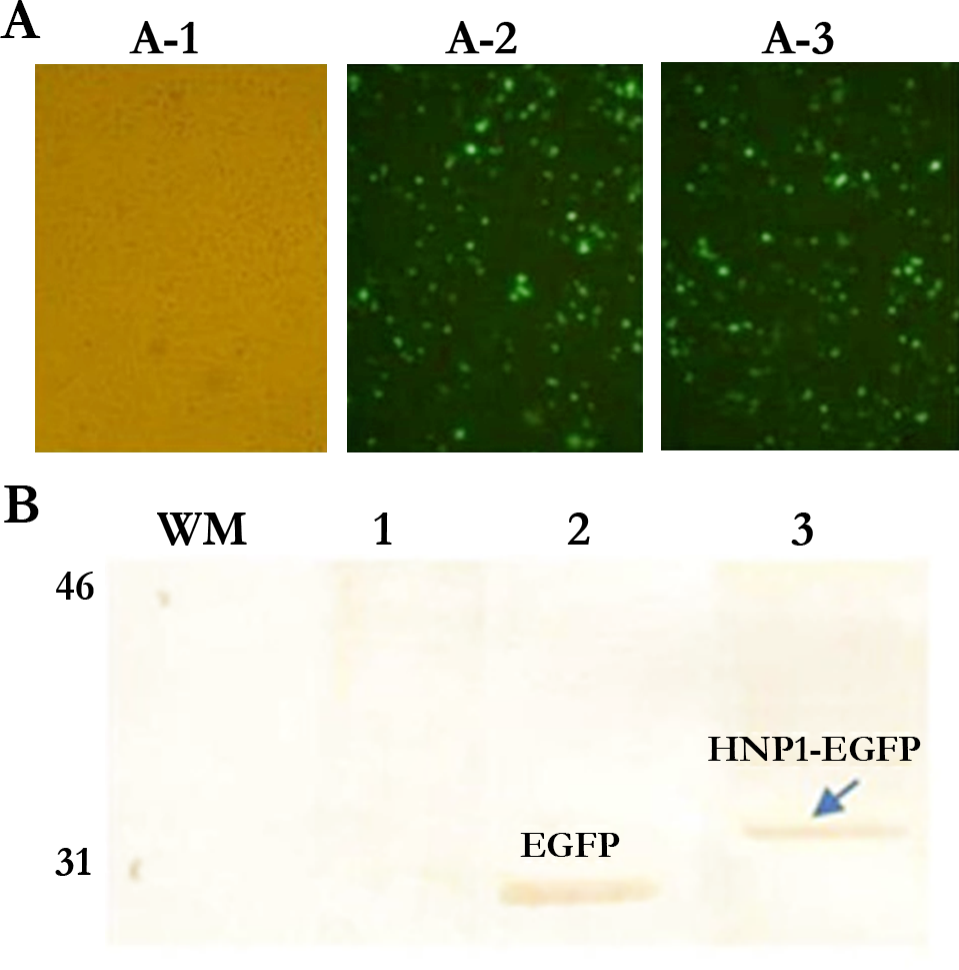
Confirmation of HNP1-EGFP transfection in COS-7 cells by fluorescence microscopy and western blot analysis. **(A)** COS-7 transfection confirmation by fluorescent microscopy 48h after transfection. A-1; Untransfected COS-7, A-2; COS-7 transfected with pcDNA-EGFP, A3; COS-7 transfected with pcDNA-HNP1-EGFP. **(B)** Confirmation of EGFP expression by western blot analysis in COS-7 transfected with pcDNA-HNP1-EGFP and pcDNA-EFGP; lane 1: untransfected COS-7, lane 2: COS-7 transfected with pcDNA-EFGP, lane 3: COS-7 transfected with pcDNA-HNP1-EFGP.

COS-7 cells were transfected by the pcDNA-HNP1-EGFP construct to confirm HNP1 production by fluorescence detection of EGFP in fluorescence microscopy and by the anti-GFP Ab in western blotting. COS-7 cells transfected by pcDNA-EGFP and the untransfected COS-7 cells were used as positive and negative controls, respectively. The expression of EGFP in pcDNA-HNP1-EGFP and pcDNA-EGFP was confirmed by fluorescence microscopy as shown in Fig 1A. By western blotting, the immunoreactive band (34.5 kD) was detected in COS-7 transfected by pcDNA-HNP1-EGFP and COS-7 transfected with pcDNA-EGFP (31 kD) (Fig 1B).

### Effect of folded HNP1 on *E*. *coli*, *L*. *major* promastigotes (IC_50_) and infected macrophage (EC_50_ determination)

**Fig 2:**
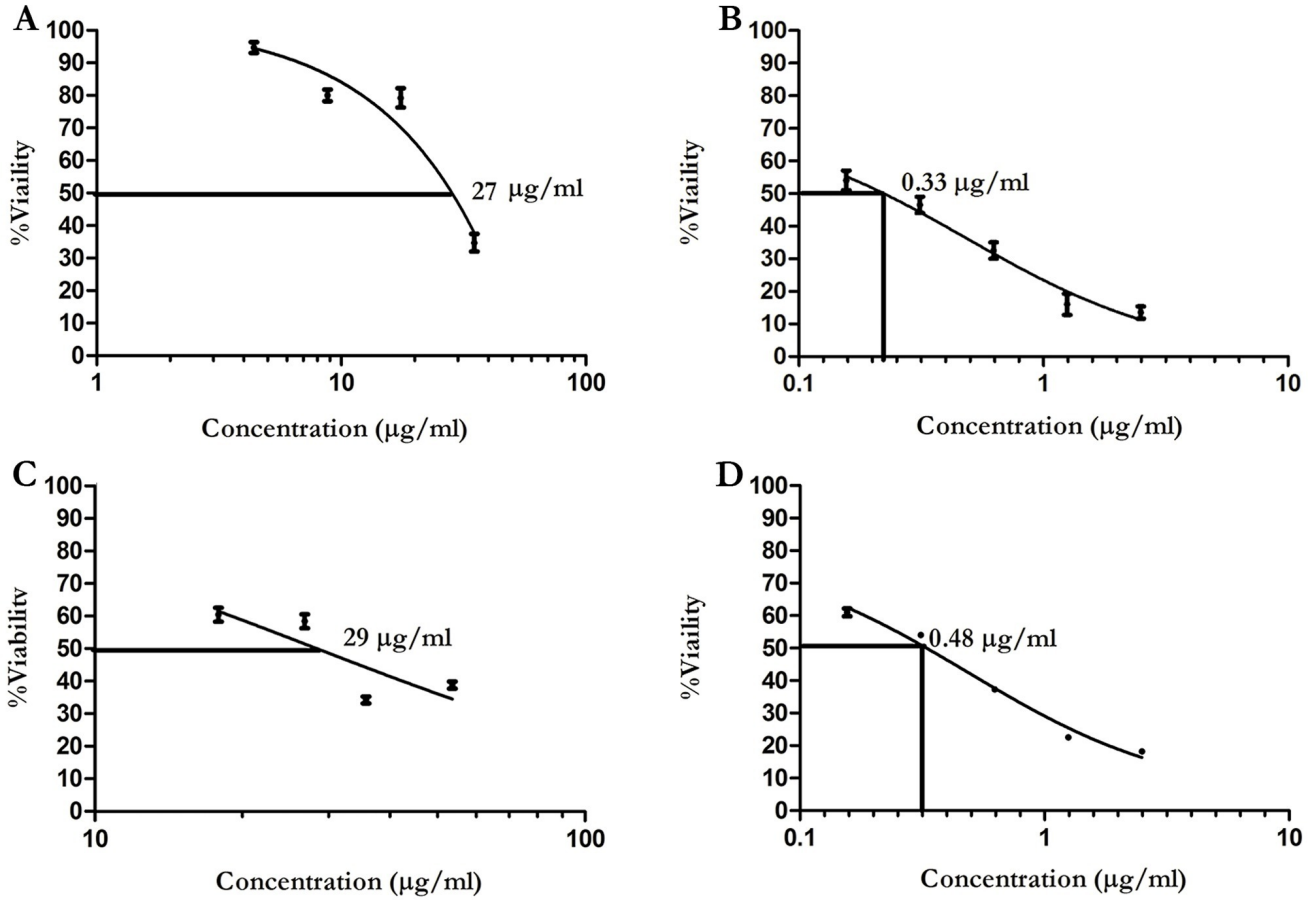
Anti promastigote and amastigote effects of folded HNP1. After HNP1 folding by oxido-shuffling method, the leishmaniacidal effect of folded HNP1 was analyzed **(A)**; the inhibitory effect of folded HNP1 on *L*. *major* promastigotes **(B)**; the inhibitory effect of AmB on *L*. *major* promastigotes **(C)**; effective dose of folded HNP1 on infected THP1 with *L*. *major* **(D)**; effective dose of AmB on infected THP-1 with *L*. *major*.

In this study, synthetic HNP1 was purchased and folded by oxido-shuffling method. To further evaluate the efficacy of folding, the killing activity was analyzed against *E*. *coli*. The colony forming unit (CFU) was lower in the folded HNP1 concentration as compared to the unfolded HNP1 with the highest inhibiting concentration of 5μg/ml. As demonstrated in S1 Table, the ratio of unfolded to folded HNP1 confirmed our observation. Based on the bacterial acquired concentration, the killing potency of the folded HNP1 was determined on logarithmic phase promastigotes (ranging from 5 to 35μg/ml). Besides, the AmB (0.15 to 2.5μg/ml) was used as an internal control [30]. The IC_50_ results indicated that the folded HNP1 inhibits promastigotes growth at 27 μg/ml compared to AmB with much lower effective dose of about 0.33 μg/ml. Based on these results the EC_50_ concentration was determined and indicated that amastigotes were inhibited by the folded HNP1 at 29 μg/ml compared to AmB with an inhibitory about 0.48 μg/ml (Fig 2).

### Footpad swelling measurement and quantification of the parasite burden in the lymph node of treated and control groups

**Fig 3:**
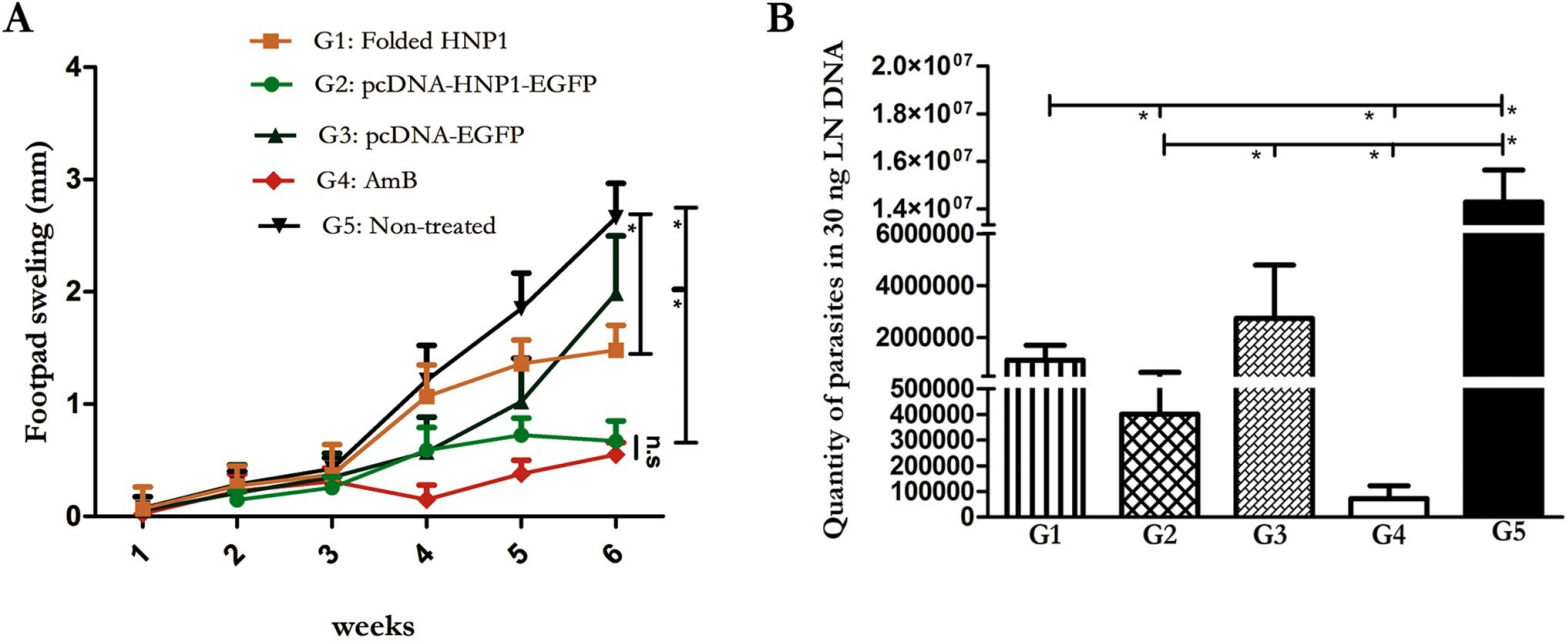
Footpad swelling measurement and determination of *L. major* parasite quantification in lymph node of treated and control groups. Footpad swelling measurement, started one week after challenge, was fulfilled weekly by metric capilar **(B)** Real time PCR was performed in 30 ng of DNA (n = 5). The G1-G5 represents the results from folded HNP1 (G1), pcDNA-HNP1-EGFP (G2), pcDNA-EGFP (G3), AmB (G4) and non-treated group (G5), respectively. The stars indicate significant difference and n.s displays not significant.

To evaluate the therapeutic effects of HNP1 with two different approaches, BALB/c mice were infected with stationary phase promastigotes in different groups. Footpad swellings were monitored weekly (started one week after and continued up to six weeks post infection). The statistical analysis at six weeks after infection showed significant difference in footpad swelling in the folded HNP1 group (G1, with determined dose as shown in S2 Fig) with the non-treated group (G5, P <0.0001) and the pcDNA-HNP1-EGFP (G2) with the pcDNA-EGFP (G3) and the G5 (P<0.0001) (Fig 3A). What is noteworthy is that the pcDNA-HNP1-EGFP treated group was able to control the footpad swelling similar to AmB (P = 0.1) which was more effective than the folded HNP1 (G1, P = 0.0031). Furthermore, the parasite burden was performed in homogenized lymph node of five mice in each group by quantitative real time PCR. As shown in Fig 3B, the treatment with either HNP1 folded peptide or pcDNA-HNP1-EGFP reduced the parasite load in the lymph node in comparison to the non-treated (G5) group (P<0.0001) quite in line with the footpad swelling. The pcDNA-HNP1-EGFP treated group controlled parasite loads better than the folded HNP1 (P = 0.0091) (similar to footpad swelling). However, the AmB treatment was more effective and significantly controlled parasite propagation in contrast to both G1 (P<0.0001) and G2 (P = 0.001). S2 and S3 Tables summarized the P-values and statistical differences among groups.

### Immunotherapy approaches induce Th1 response

**Fig 4:**
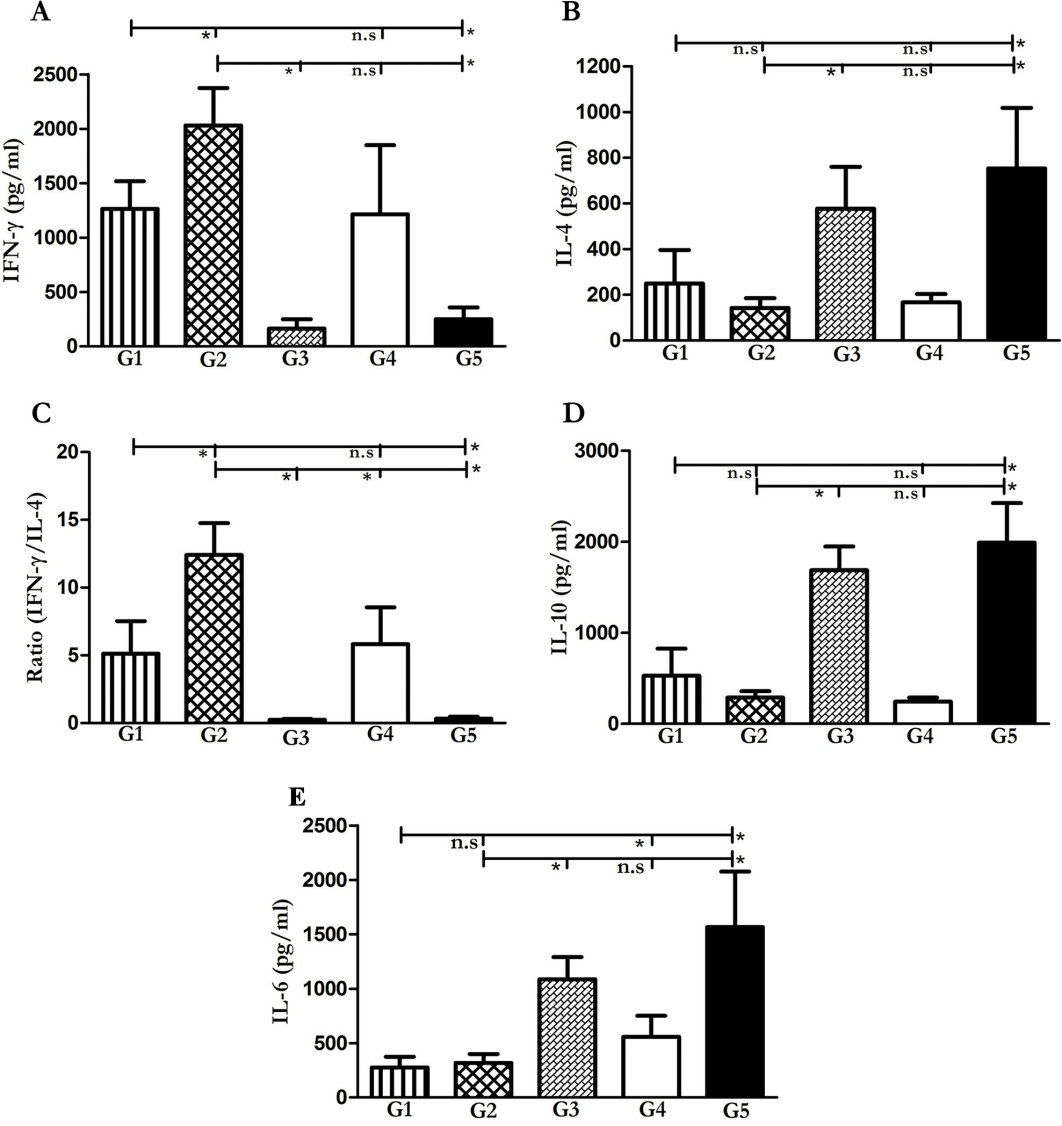
Cytokine production by splenocytes stimulated with F/T *L. major* antigen in treated and control mice. Five mice per group were sacrificed and their spleens were dissected and stimulated by F/T antigen in order to measure IFN-γ **(A)**, IL-4 **(B)**, IFN-γ/IL-4 ratio **(C)**, IL-10 **(D)** and IL-6 **(E)** cytokine production by ELISA system. The G1-G5 represents the results from folded HNP1 (G1), pcDNA-HNP1-EGFP (G2), pcDNA-EGFP (G3), AmB (G4) and non-treated group (G5), respectively. The star represents significant difference and n.s illustrates not significant.

Given that the Th1 immune response is necessary against *Leishmania* infection [31], we analyzed the level of IFN-γ, IL-4 and, IL-10 in splenocytes re-stimulated with *L*. *major* F/T as the re-call antigens. Six weeks after infection, five mice from each group were sacrificed and spleens were dissected and homogenized for different cytokine measurements. As shown in Fig 4, both therapeutic approaches folded HNP1 (G1) and pcDNA-HNP1-EGFP (G2), induced high levels of IFN-γ, P<0.0001 and low levels of IL-4 (P = 0001 and P = 0002 respectively) compared to the non-treated group (G5). Meanwhile, there was a difference between G2 and pcDNA-EGFP (G3) groups so far as IFN-γ and IL-4 were concerned (P<0001). Higher IFN-γ/IL-4 ratio and lower IL-4 and IL-10 in both the groups demonstrated the Th1 polarized immune response by these two approaches (G1, G2) while the ratio was too low in G5 and G3 groups.

Comparing the results between the pcDNA-HNP1-EGFP treated group and the folded HNP1 one, it is clear that the pcDNA-HNP1-EGFP treatment induced significantly higher levels of IFN-γ/IL-4 and lower levels of IL-4 and IL-10 as shown in Fig 4A, 4B, 4C and 4D. Interestingly, the AmB (G4) similar to G1 and G2 treated groups, produced comparable high levels of IFN-γ and IFN-γ/IL-4 and low levels of IL-4 and IL-10 cytokine. In this study, the level of IL-6 was also analyzed as a pro-inflammatory cytokine. Both folded HNP1 and pcDNA-HNP1-EGFP showed significantly lower levels of IL-6 in comparison to the non-treated group (P<0001). IL-6 level was significantly higher in the AmB treated group compared to the folded HNP1 (P = 0.0007) and no significant difference was seen in respect to the pcDNA-HNP1-EGFP treated group (P = 0.02). IL-6 production in both HNP1 and pcDNA-HNP1 groups (G1 and G2) were statistically non-significant (P = 0.4). S2 and S3 Tables summarize all P-values and statistical differences between groups

### Decreased arginase activity and increased NO generation in mice treated with folded HNP1 and pcDNA-HNP1-EGFP

**Fig 5:**
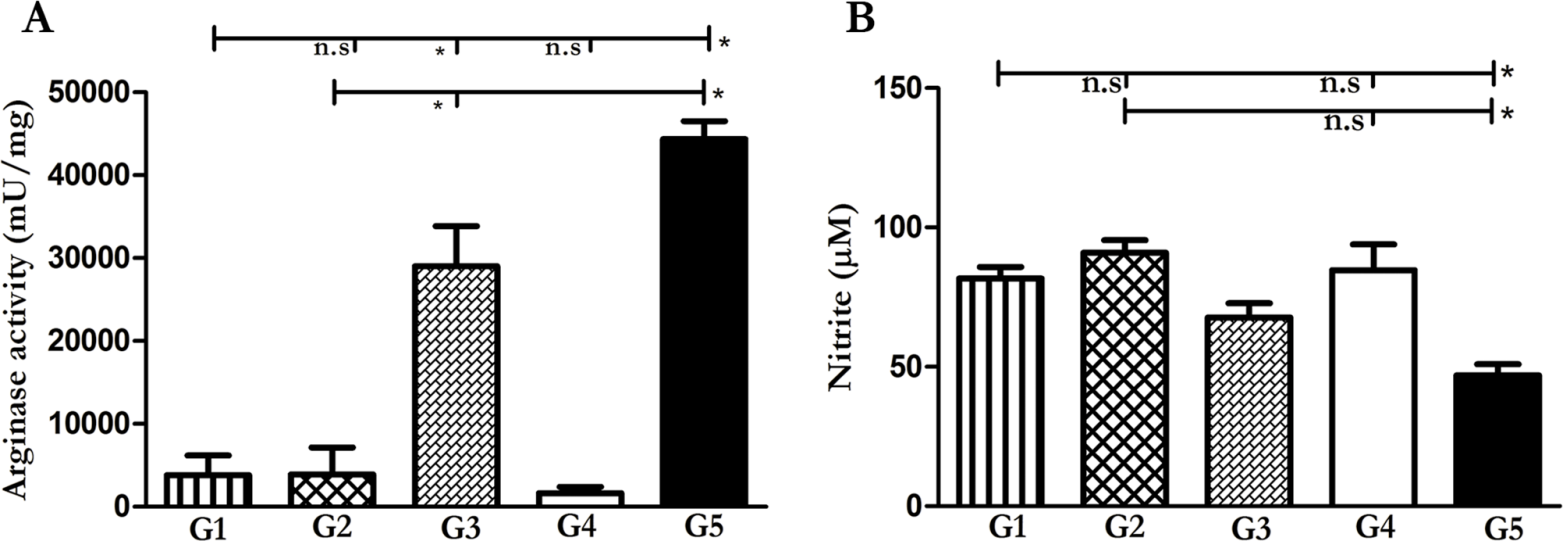
Evaluation of arginase activity and NO production in mice treated with different HNP1 modalities. Arginase activity (mU/ml) and nitrite production were determined in footpad and spleen (n = 5), respectively. Arginase activity **(A)** was performed by micro method test and NO **(B)** was fulfilled by Griess test. In both tests, the G1-G5 represents the results from folded HNP1 (G1), pcDNA-HNP1-EGFP (G2), pcDNA-EGFP (G3), AmB (G4) and non-treated group (G5), respectively. The star displays significant difference and n.s indicates not significant.

L-Arg is metabolized either by arginase or iNOS based on the signal from the micro-environment. Th1 milieu promotes the expression of iNOS and NO generation while the Th2 cytokines increase the expression of arginase enzyme [32]. As shown in Fig 5, both the therapeutic approaches (G1 and G2) significantly increase the anti-*Leishmania* nitric oxide level compared to the non-treated group G5, respectively. This is in line with the increased IFN-γ production and the induced Th1 milieu by both the folded HNP1 (G1) and the pcDNA-HNP1-EGFP (G2). As expected, arginase activity was inversely reduced in both the treated groups (G1 and G2) when compared to the non-treated group (P = 0.0006 and P = 0.0002, respectively). Furthermore, there was no significant difference between nitrite production and arginase activity of both the treated groups when compared with each other (P = 0.149 and 0.72 respectively). The same result was observed in both the therapeutic approaches (G1 and G2) with AmB in nitrite production (P = 0.75 and P = 0.1 respectively) and the arginase activity was compared to the AmB (P = 0.013 and P = 0.76) treated group, which means that both the approaches were able to induce Th1 milieu comparable to AmB as a current therapy. Our results showed that G5 had the highest arginase activity and the G3 group, the lowest NO production. S2 and S3 Tables summarize all P-values and statistical differences between groups.

### HNP1 treatment leads to partial improved histopathology in BALB/c mice

**Fig 6:**
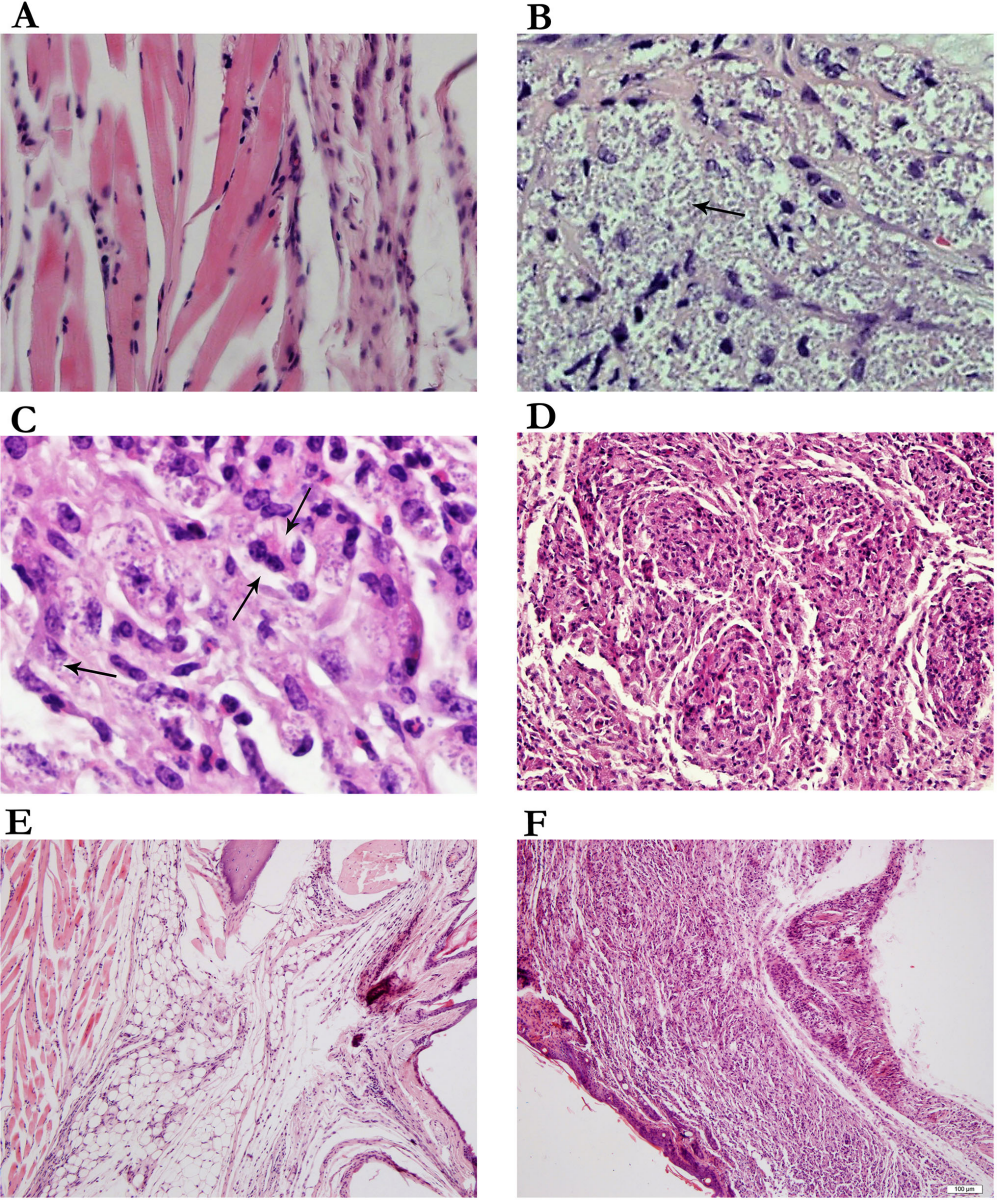
Histopathological changes in different mice groups. Histopathological changes were analyzed directly on the infected footpads **(A and B)** Presence of parasites in the footpad lesion which represent; **A**: No parasite, **B**: parasite 4(+) **(C)** Presence of plasma cell in non-treated group (G5) **(D)** Immature granuloma reaction. Inflammation assessment **(E)**: restricted area inflammation in treated group with HNP-1 and **(F)**: diffuse inflammation in non-treated group was observed. (H&E, 400 HPF for Fig A, B, C. 250 for Fig D and 40 for Fig E and F).

In this study, histopathological changes were analyzed directly in the inflamed footpad. The parasite load for each footpad was graded from; Not Seen (NS) to 4(+) as illustrated in Fig 6A and 6B. The folded HNP1 treated group (G1) was graded from rare to minor the pcDNA-HNP1-EGFP (G2) group was graded from not seen to rare in respect to parasite load. These results were likely for the AmB treated group in parasite load. Instead, the non-treated group (G5) was graded as severely indicating a remarkable number of parasites (Table 2). Interestingly, in all the folded HNP1, pcDNA-HNP1-EGFP and AmB treated groups limited inflammation was present in the injection site in contrast to the diffused inflammation in the non-treated group (Table 2). Plasma cells (Fig 6C) and immature granuloma (Fig 6D) were only seen in the non-treated group. Fig 6E and 6F represent in limited and diffused inflammation, respectively. There was no significant difference in neutrophil infiltration in different groups. In our experiment, the major histopathological alteration was related to the G5 group and after that, the G3 group regarding highest parasite load and diffused inflammation. Table 2 summarizes all histopathological changes observed in different treated and control groups.

**Table 2:** Dermal changes in the infected footpad of BALB/c mice in treated and control groups.

Group	Parasite	Plasma cell	Granuloma	Inflammation
G1-1	N.S *	-	-	0–1 (limited)
G1-2	2(+)	-	-	0–1 (limited)
G2-1	N.S	-	-	0–1 (limited)
G2-2	1(+)	-	-	1–2 (limited)
G3-1	2(+)	-	-	2(+) (limited)
G3-2	2(+)	-	-	3(+) (diffuse)
G4-1	2(+)	-	-	2(+) (limited)
G4-2	2(+)	-	IG**	2(+) (limited)
G5-1	4(+)	+	IG	4(+) (diffuse)
G5-2	4(+)	+	IG	4(+) (diffuse

Two mice were euthanized at six weeks post infection and their lesions were fixed in formalin for histopathological analysis. G1: folded HNP1, G2: pcDNA-HNP-EGFP, G3: pcDNA-EGFP, G4: AmB, G5: non-treated.

*N.S: Not Seen

**IG: Immature Granuloma

## Discussion

Despite several efforts for providing a proper vaccine against leishmaniasis, there is no approved vaccine for humans yet [33]. At the moment, the only approach for parasite control is chemotherapy [8]. Increased antibiotic resistance and high toxicity to first and second line drugs have forced the use of more effective drugs with less toxicity. Besides, some of drugs such as miltefosin are limited because they are not cost effective [31,34]. Various study showed the role of Th1 response in leishmaniasis recovery, so the researchers have applied the immunomodulators and effective cytokines to boost immune response for leishmaniasis healing [33,35]. Several pieces of evidence demonstrated the role of defensins, particularly HNP1, in innate (as an alarmin molecule) and adoptive immunity (i.e. increase of dendritic cell maturation) [36,37], but there are a few reports of antimicrobial peptide effects on *Leishmania* spp [4].

In this study, we aimed to evaluate the therapeutic effect of HNP1 as an antimicrobial peptide with immunoregulation potential and direct killing activities. To this end, the synthetic HNP1 was compared to the AmB as current therapy and also the pcDNA expressing system. HNP1 is a 30- amino-acid-long peptide which is fully active when properly folded by covalent bindings between cysteine. So, folding is the pre-requisite of HNP1 action produced synthetically or in recombinant form. Here, we used the oxido-shuffling (the suitable method for folding of cysteine rich peptides) and analyzed the *in vitro* killing activity against *Leishmania* parasite (both promastigote and amastigote) before starting the treatment in mice. The *in vivo* concentration of AmB for parasite killing was higher than *in vitr*o leishmaniacidal effect of amphotericin B. It may have caused the *in vitro* killing of AmB limited to resident infected macrophages, while *in vivo* leishmaniacidal effect of AmB depends on Th1 cell type network [38]. In different studies, AmB is used in combination with immunomodulators such as HNP1 and IL-12 in candidiasis and visceral leishmaniasis, respectively [38,39]. Murray *et al*. showed that the killing effect of AmB is 7.5-fold more when combined with IL-12 against visceral leishmaniasis in mice model [38]. Here, we showed similar action for the two HNP1 approaches and they are almost similar to the AmB but not for the lymph node parasite load. Opposite the highlighted role of the AmB in free radical production resulted in autoxidation and binding to sterol and pore formation [40,41], the immunoregulation of HNP1 as an AMP member is highlighted [10]. Meanwhile, some studies described that the *in vitro* MIC (minimum inhibitory concentration) of the AMP against microbes are higher than the effective concentrations found *in vivo* [9]. However, we showed that the *in vitro* and *in vivo* doses of HNP1 on *L*. *major* parasites were almost equal. Although this peptide has anti-protease activity, there is little knowledge about it [42]. Besides, the folding process is very difficult and providing the folded peptide is not cost effective. Here, to further explore more applicable options, the DNA expressing HNP1 as an immunotherapeutic vaccine was used concurrently with the folded HNP1.

It is worth mentioning that we used pcDNA as a therapeutic tool due to safety, easy production with high purity, non-replication and prolonged expression of encoded gene. DNA-based vaccines have been considered as impressive remedial procedures for malignancies [43,44]. Besides, these vectors contain CpG motifs as adjuvant that induces Toll-like receptor (TLR-9) [45]. Unlike common adjuvants (oil based) which can elicit Th2 response, CpG oligodeoxy nucleotide (ODN) can elicit mostly Th1 response by generating IgG2a and IFN-γ in splenocytes, [46–49]. This plasmid can enter the APC and stimulate the CTL response as well as the Th1 response. Furthermore, multiple plasmid DNA administrations do not stimulate anti-plasmid autoimmunity, so multiple delivery is well tolerated [50]. The induced immunity by pcDNA-HNP1-EGFP showed that this approach can stimulate Th1 immune response like the folded HNP1 with higher IFN-γ and IFN-γ/IL-4 and even it was better than the AmB in IFN-γ production and the IFN-γ/IL4 ratio. This result is in agreement with other reports in LL-37/hCAP18 which said gene delivery has more effect compared to synthetic peptide [51,52]. Jacobsen *et al*. showed that hCAP-18/LL-37 adenoviral delivery in infection due to *P*. *aurogonisa* after burn wounds could be up to 1,000-fold stronger compared to the therapeutic effect of synthetic LL-37 peptide [51]. Besides, in their last studies, they concluded that EGFP cannot stimulate immune response and it is seen that this response is related to pcDNA [53].

Our *in vivo* results showed that both the approaches effectively reduced the parasite load much more than the non-treated group. However, it is more pronounced in pcDNA-HNP1-EGFP compared to the folded HNP-1. Our data illustrated that, the folded HNP1 and the pcDNA-HNP1-EGFP can induce IFN-γ and nitric oxide generation as a credible Th1 immune response in comparison to the non-treated group. It is completely clear that the killing of *Leishmania* parasite was not only dependent on superoxide but also NO_2_ production which was generated by M1 macrophages [54,55]. In this experiment, decrease of parasite load associated with IFN-γ and NO generation (as cellular immune response) can help heal leishmaniasis[56]. Our results, showed that the highest arginase activity and IL-4 and IL-10 production was in the non-treated group compared to other groups. In various infectious, diseases such as leishmaniasis, trypanosomiasis and viral infection, are concordance with IL-4 as well as IL-10 production (as Th2 cytokines) and L-Arg increased catabolism in M2 macrophages [57,58]. The IFN-γ/IL-4 ratios in folded HNP1 and pcDNA-HNP1-EGFP were manifold higher than the non-treated group. Our findings are in agreement with other studies describing the IL-4 production driven Th2 response which has been demonstrated to facilitate non-healing response [59,60].

The severity of cutaneous leishmaniasis depends on the rate of parasite load and the diffused inflammatory reaction at the site of infection. So, tissue pathology is an important evaluation in order to precisely determine the effect of therapy on the infected site. BALB/c mice are very susceptible where widespread damage, inflammation and parasite replication are commonly observed. In our study, the visualization of high rate of parasites was associated with the unorganized granuloma and plasma cell observation only in the non-treated group. This reflected uncontrolled (diffuse) inflammation in contrast to limited and controlled inflammation in all the treated groups (G1, G2 and G4) [61,62]. These results are in tandem with other studies. This implies that immature granuloma was seen in patients whose CL initial diagnosis without therapy and with immature granuloma was directly detected with parasite load [62]. In the non-treated group, plasma cell infiltration and immature granuloma are the reflection of the Th2 response. This is consistent with the high rates of parasites (G3 and G5). On the contrary, the limited inflammation as well as the low level of parasite, and the lack of plasma cell can anticipate Th1 response and parasite elimination as seen in G1, G2 and G4 [62]. In both the HNP1 formulation, there were decreased parasite and inflammation in pathological analysis similar to the AmB treated group. Furthermore, the investigation revealed low rate of IL-6 production in both HNP1 formulations compared to the non-treated group. IL-6 is an important pleiotropic cytokine in the expansion of Th1 or Th2 immune response [63]. IL-6 activity at a low level is essential to balance both Th17 and Treg functions [64]. In the AmB treated group, increased footpad inflammation was consistent with the IL-6 level. Anyway, the highest rate of inflammation in the non-treated group correlated well with the highest IL-6 level in this group.

Nowadays, it is clear that immunotherapies have emerged as a powerful treatment option in different clinical manifestations such as cancer in two fundamental aspects such as long term immunity against tumor and to prevent cancer recurrence [65]. Nevertheless, immunotherapy is a new term for neglected diseases such as leishmaniasis. It is necessary to re-evaluate the chemotherapy approaches and combine this approach with other immunotherapeutic approaches such as antimicrobial peptides. Despite the hallmark characteristics of antimicrobial peptides, there are few *in vivo* experiments about the effect of antimicrobial peptides on leishmaniasis [4]. In this study, we showed that in both treatment modalities by HNP1, the Th1 response potentially controls the infection compared to the non-treated group, despite parasite match with AmB. HNP1, as an immune-enhancer, can probably lower the effective dose of AmB by raising the potential immune response. The folding of this peptide is a hard task to achieve, researchers have examined the truncated analogs without disulfide bonds which can raise the potential of HNP1 for therapeutic application [66]. Besides, some studies presented a combinatory gene therapy, which stimulated synergistic effect by activating several pathways of the immune system. This could be considered in further experiments [67–69]. In our study, the pcDNA-HNP1-EGFP was a potential contributor to the combinatorial therapy with AmB.

## Supporting information

S1 Table Antibacterial activity of folded HNP1 peptide as compared to its unfolded form. The ratio is represented of unfolded to folded HNP1 activity against *E*. *coli*.
(DOCX)

S2 Table P values differences of clinical and immunological tests between G1 and other groups. (DOCX)

S3 Table P values differences of clinical and immunological tests between G2 and other groups. (DOCX)

S1 Fig The purity degree of commercialized HNP1 synthesis. (TIF)

S2 Fig *In vivo* dose determination of folded HNP1 in different concentration. (TIF)
