# Supplementary material for: Human Neutrophil Peptide 1 as immunotherapeutic agent against Leishmania infected BALB/c mice
Source: PLoS Negl Trop Dis. 2017 Dec 18;11(12):e0006123. doi: 10.1371/journal.pntd.0006123 (PMC5749894; doi:10.1371/journal.pntd.0006123)
Supplement: S1 Table — The ratio is represented of unfolded to folded HNP1 activity against E. coli. (DOCX) [file pntd.0006123.s001.docx]

| **concentration** | **Unfolded/folded ratio** |
| --- | --- |
| 2.5 µg/ml | 4.6 |
| 5 µg/ml | 6.1 |
| 10 µg/ml | 1.8 |
| 20 µg/ml | 1.6 |
| 30 µg/ml | 1.6 |
| 40 µg/ml | 2.9 |
