# Supplementary material for: Human Neutrophil Peptide 1 as immunotherapeutic agent against Leishmania infected BALB/c mice
Source: PLoS Negl Trop Dis. 2017 Dec 18;11(12):e0006123. doi: 10.1371/journal.pntd.0006123 (PMC5749894; doi:10.1371/journal.pntd.0006123)
Supplement: S2 Table — (DOCX) [file pntd.0006123.s002.docx]

|  | G1 & G2 | G1& G4 | G1&G5 |
| --- | --- | --- | --- |
| Footpad swelling | P= 0.0066 | P= 0.0031 | P <0.0001 |
| Parasite load | P= 0.0091 | P <0.0001 | P <0.0001 |
| Arginase activity (mU/mg) | P= 0.72 | P= 0.0136 | P= 0.0006 |
| IFN-γ (pg/ml) | P= 0.0063 | P= 0.85 | P <0.0001 |
| IL-4 (pg/ml) | P= 0.676 | P= 0.33 | P= 0.0011 |
| IFN-γ/IL-4 | P: 0.474 | P= 0.573 | P <0.0001 |
| IL-10 (pg/ml) | P= 0.029 | P= 0.0004 | P= 0.0009 |
| IL-6 (pg/ml) | P= 0.4 | P= 0.0007 | P <0.0001 |
| NO (µM) | P= 0.149 | P= 0.75 | P <0.0001 |
|  |  |  |  |

Two- tailed parametric and non-parametric test, significance level was adjusted according to Bonferroni method (in case of multiple comparison)
