# Supplementary material for: Human Neutrophil Peptide 1 as immunotherapeutic agent against Leishmania infected BALB/c mice
Source: PLoS Negl Trop Dis. 2017 Dec 18;11(12):e0006123. doi: 10.1371/journal.pntd.0006123 (PMC5749894; doi:10.1371/journal.pntd.0006123)
Supplement: S3 Table — (DOCX) [file pntd.0006123.s003.docx]

|  | G2& G3 | G2& G4 | G2 &G5 | G3 & G5 |
| --- | --- | --- | --- | --- |
| Footpad swelling | P <0.0001 | P= 0.1 | P <0.0001 | P= 0.053 |
| Parasite load | P= 0.01 | P= 0.001 | P <0.0001 | P <0.0001 |
| Arginase activity (mU/mg) | P <0.0001 | P= 0.76 | P= 0.0002 | P= 0.034 |
| IFN-γ (pg/ml) | P <0.0001 | P= 0.006 | P <0.0001 | P= 0.023 |
| IL-4 (pg/ml) | P <0.0001 | P= 0.058 | P <0.0002 | P= 0.143 |
| IFN-γ/IL-4 | P= 0.0002 | P= 1 | P <0.0001 | P= 0.075 |
| IL-10 (pg/ml) | P <0.0001 | P= 0.247 | P <0.0001 | P= 0.113 |
| IL-6 (pg/ml) | P <0.0001 | P= 0.02 | P= 0.0002 | P= 0.015 |
| NO (µM) | P <0.0001 | P= 0.1 | P <0.0001 | P= 0.005 |
|  |  |  |  |  |

Two- tailed parametric and non-parametric test, significance level was adjusted according to Bonferroni method (in case of multiple comparison)
