# Supplementary figures and images for: Human Neutrophil Peptide 1 as immunotherapeutic agent against Leishmania infected BALB/c mice
Source: PLoS Negl Trop Dis. 2017 Dec 18;11(12):e0006123. doi: 10.1371/journal.pntd.0006123 (PMC5749894; doi:10.1371/journal.pntd.0006123)

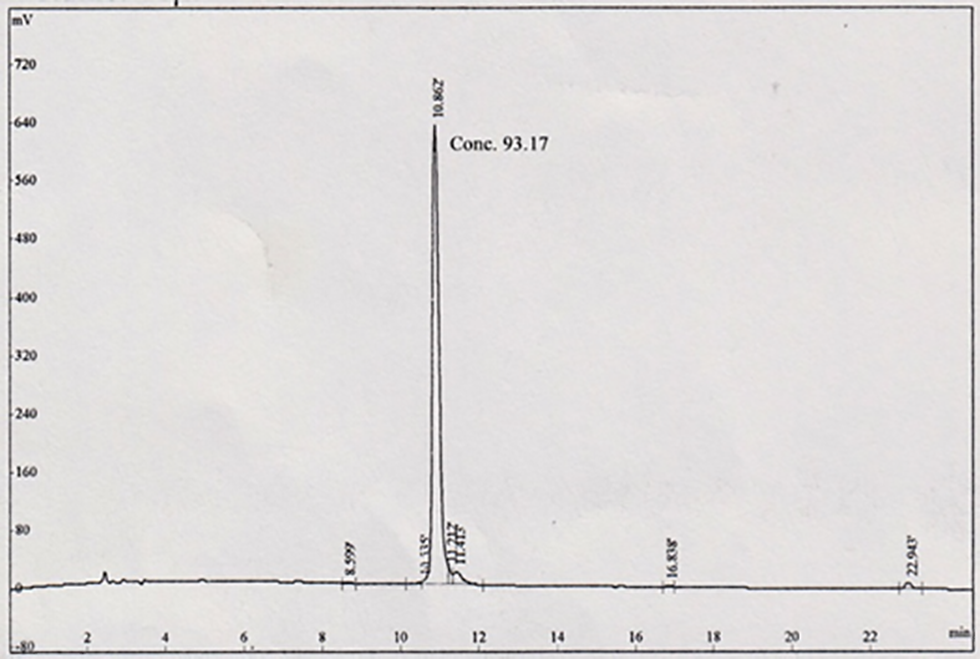

Supplement: S1 Fig — (TIF) [file pntd.0006123.s004.tif]

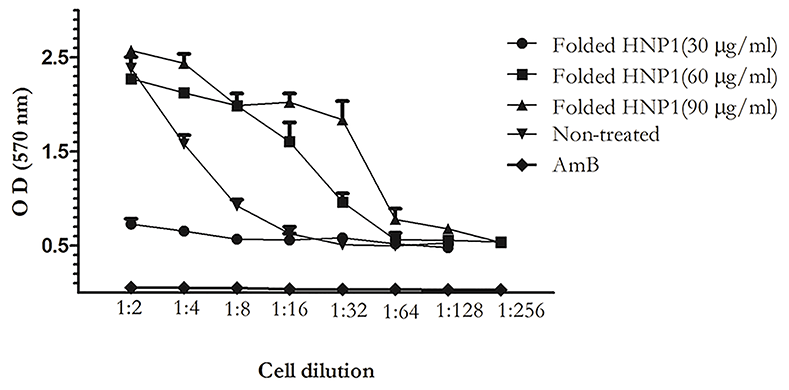

Supplement: S2 Fig — (TIF) [file pntd.0006123.s005.tif]
